# The role of emergency medicine physicians in trauma care in North America: evolution of a specialty

**DOI:** 10.1186/1757-7241-17-37

**Published:** 2009-08-23

**Authors:** Michael D Grossman

**Affiliations:** 1 St. Luke's Hospital, Trauma and Surgical Critical Care, Estes Surgical Group 801 Ostrum Street, Bethlehem, PA 18015, USA

## Abstract

The role of Emergency Medicine Physicians (EMP) in the care of trauma patients in North America has evolved since the advent of the specialty in the late 1980's. The evolution of this role in the context of the overall demands of the specialty and accreditation requirements of North American trauma centers will be discussed. Limited available data published in the literature examining the role of EMP's in trauma care will be reviewed with respect to its implications for an expanded role for EMPs in trauma care. Two training models currently in the early stages of development have been proposed to address needs for increased manpower in trauma and the critical care of trauma patients. The available information regarding these models will be reviewed along with the implications for improving the care of trauma patients in both Europe and North America.

## Introduction

The role of Emergency Physicians (EMP) in the care of trauma patients varies considerably depending upon locale, available expertise and presence or absence of regulatory agencies. This review attempts to define some of these variables and suggests alternative models based upon existing needs and resource availability. Much of the discussion is centered on the North American experience that has evolved over the past forty years. There may be certain features applicable to European systems which themselves have significant variability between countries. A fundamental assumption underlying this review is that there should be measurable standards of care for trauma patients and that these will include some degree of expertise and perhaps even specialization in trauma care. This field has come to be known in the US as "Traumatology" in distinction to Trauma Surgery. Whether expertise in Traumatology is the province of surgeons, EMP's, or other physicians remains a matter of discussion in North America and there is little data available addressing the question. Similarly, training models for EMPs that are in place in Europe differ from those in the US and thus the relative skill sets with respect to trauma care are difficult to compare. Newer models in North America that emphasize advanced training in trauma and critical care for EMP's are just being developed and have not been well studied.

## Workflow considerations

Emergency department (ED) workflow is governed by patient volume and staffing. Busy ED's in North America may see more than 100,000 patients per year and therefore several hundred visits per-day. Data regarding volume per provider may vary depending on staffing patterns but generally should not exceed 2–3 patients per provider per hour [1]. In busy ED's this goal may be difficult to achieve. There is considerable literature dealing with the subject of overcrowding and its causes [2,3]. Most authors agree that a significant percentage of ED volume consists of low-acuity "walk-in" disease that has increased as primary care networks become more difficult to access, and while the overall affect of low acuity patients on overcrowding is thought to be nominal [2,3], it may impact physician work flow. In addition, in many Emergency Departments in the US, the EMP serves a gatekeeper function with responsibility for the initial phase of care for all acute care emergencies admitted to the hospital. Recent data indicates that an increasing percentage of all hospital admissions (18–25%) in the US come through the ED, thus patients may be "held" in the ED awaiting admission but in many cases must still be cared for by EMP's. Finally, in some hospitals, EMP's are required to leave the ED in order to respond to hospital emergencies as members of a code or rapid response team further affecting prioritization and work flow considerations.

All these factors impact work flow for the EMP. Since most major trauma centers in North America are associated with busy ED's the added burden of managing complex trauma patients is a consideration affecting the involvement of all ED staff including physicians. The American College of Surgeons Committee on Trauma (ACSCOT) handbook on optimal care of the injured patient specifically states that "The Emergency physician may be assigned control [of the trauma patient] until the surgeon arrives" Since the expectation is that a surgeon will respond within five minutes in Level I and II trauma centers, participation by the EMP beyond that time period is not required, at least by ACSCOT standards [4]. Those standards go on to describe that following arrival of the surgeon; performance of diagnostic and therapeutic procedures will be "shared in accordance with mutually agreed upon protocols". Data published more than ten years ago demonstrate that the average time per-patient required to care for all blunt trauma patients is approximately one hour per patient and that severely injured patients may require 6–8 hours of continuous care [5]. Thus the overall impact of caring for trauma patients on work flow considerations in US ED's may vary considerably depending on the role of the EMP relative to the trauma team.

Current models of trauma care in the US frequently utilize an approach that brings the trauma team to the ED, often in a somewhat separate physical space, and usually involves the EMP for some phase of resuscitation, then returns the EMP to the department. This appears to be least disruptive to work flow in large departments with busy trauma centers. In Canada where EMP's, Anesthesiologists or Surgeons may function as trauma team leaders, the Trauma Association of Canada still requires that trauma call covered by EMP's must be kept separate from general ED duties so that there are no conflicts between ED work flow and trauma care [6,7].

In ED's with lower volumes, workflow patterns may be more favorable with respect to the competing priorities described above. However, these ED's may be associated with hospitals that do not possess the specialty care required for trauma patients after the resuscitative phase of care is completed. In the US, EMP's in these lower volume departments may be more likely to perform initial resuscitative care and transport the patient to a trauma center. EMP's therefore may play a significant role in the initial phase of trauma care as part of their overall work flow in many US hospitals that are not trauma centers. The timeliness and adequacy of care by EMP's under these circumstances is most often not well monitored since trauma care in these hospitalized is not necessarily scrutinized by ACSCOT trauma center accreditation standards.

## Historic considerations: US model for trauma care

The concept of regionalized civilian trauma networks in the United States dates back at least to the late 19^th ^century when William Estes created a system of care for injured railroad workers and miners in Northeast Pennsylvania [8]. The formation of a committee to study outcomes in fracture management of which Dr Estes was a founding member gave rise to what would become the American College of Surgeons Committee on Trauma (ACS-COT).

Beginning with the study by Zollinger in 1955, a number of publications over the next 30 years examined the concept of preventable trauma deaths [9-12]. Several landmark studies related preventable deaths to hospital size (defined as "hospital category") and the presence or absence of a regional trauma hospital [13-15]. Reacting at least in part to data provided by these studies the ACSCOT initially published guidelines for staffing and organizing a "Trauma Center" in 1976 [4]. This publication has undergone multiple revisions and re-editions but remains the standard document describing the resources required for optimal care of trauma patients.

As early as 1966 the role of physicians staffing emergency rooms in the care of trauma patients was discussed as an important element in preventable trauma death [16]. The publication by Lowe et al in 1983 documented specific opportunities for improvement in preventable trauma death that were related to timeliness of care and appropriate diagnosis and treatment provided by emergency rooms in hospitals in the state of Oregon [17].

The impetus to develop a specialty of emergency medicine in the US was driven in part by issues related to emergency care for trauma patients [10,16-19]. Generalists or family physicians staffed North American ED's and specialists were called to manage and admit patients according to need. In this regard, North American ED's of the 1960's and 1970's resembled current practice in some European countries. Based in large part upon the sentinel study published in 1966 the ACSCOT supported the concept of specialized physicians with training in Emergency Medicine but maintained the primary role of surgeons as "captain of the ship" in trauma care [4,16]. Current standards for Level I trauma center accreditation in the US require 24 hour emergency room coverage by a trained EMP and immediate availability of surgeons who must be present within 5 minutes of the arrival of the trauma patient.

The degree to which surgeons and EMP's interact in the care of trauma patients and the specific clinical roles of each provider is addressed by the ACSCOT and is dependent upon the level or complexity of the trauma center. As noted previously, for trauma centers with level I or II designations, a surgeon must be immediately available with full time EM staff assigned control of the trauma patient until the surgeon arrives. In addition, the standards for optimum care describe specific physiologic, anatomic and mechanistic criteria that should mandate the highest level of trauma response within a trauma center that would require the surgeon to be present.

## Challenges to the exisiting model for optimum care of the trauma patient

From its inception there have been numerous challenges to implementation of the standards for optimal care of the trauma patient. Trunkey and West identified these issues 30 years ago commenting that "...staffing requirements are stringent and for the most part can only be met in a large university teaching hospital with house staff" [15]. In addition, the standards do not address the larger issue of whether or not hospitals may choose to participate in a state or regional trauma system. Thus in many regions of the US patients may be delivered to a hospital that does not participate in a trauma system and therefore may not possess the resources for optimal care.

Even within the established, well-developed trauma networks present in large urban and suburban centers within the US and Canada there are many challenges to providing optimal trauma care. It is because of these issues that new and different roles for EMPs in trauma care have been suggested. Examples of challenges to the ideal model envisioned by the ACS-COT include an overall decline in surgical manpower, a declining interest among surgeons in providing trauma care, and a shift in access patterns for health care that has placed increasing burdens on emergency departments and availability of specialists to cover emergency call [20-25].

Optimal care of trauma patients involves more than the resuscitative phase of care carried out in the emergency department and includes critical care, surgical specialty care, rehabilitation and follow-up. Additional challenges exist in several of these areas; those that involve the ICU phase of care may affect the role of EMP's in trauma care [26,27]. Recent publications have cited inconsistencies between ASCOT standards for participation of trauma surgeons in the critical care of trauma patients and the realities of practice in US trauma centers [27]. Lack of manpower among Surgical Intensivists/Trauma Surgeons has resulted in the increased use of non-surgeon intensivists including EMP's who have received additional training in critical care [26]. This environment more closely resembles models in place in Europe where non-surgeon intensivists are the most common care providers for trauma patients in the ICU.

## Role of EMP'S in trauma care

The role of EMP's in trauma care in North America varies considerably and is dependent upon whether or not the ED is associated with a trauma center, the level of trauma center designation, the presence or absence of regulatory guidelines and the nature of those guidelines. In addition, the presence or absence of surgical back-up, critical care resources and surgical specialty care will affect the hospitals' ability to care for trauma patients and therefore the role of EMP's. Finally, EMP training and experience will affect willingness and ability to care for trauma patients.

Residency training in Emergency Medicine in the US requires a minimum of 36 months of training in an accredited residency. Training must include the performance of 35 adult trauma resuscitations but there is no provision for determining how the resident will be assured a role as team leader in those resuscitations. Two months of dedicated critical care on a separate inpatient service are also required. There is no requirement for rotation on a trauma service [28]. There might be considerable variation in training with some programs including 2–3 months of rotations on a trauma service and/or separate critical care rotations. Residents from these programs might have a higher comfort level with trauma care than those whose training allows exposure to trauma only as part of their general EM training, particularly if a separate trauma team responds for trauma activations.

In smaller North American hospitals that are not designated as trauma centers the first, and often the only physician responder for trauma patients will be most likely an EMP. Though no formal data exist it is likely that many of these hospitals where care is rendered exclusively by EMPs are smaller hospitals with limited surgical capability and fewer ED visits where the arrival of a trauma patient is likely to be less disruptive to the overall function of the Emergency Department. In an organized regional trauma system any seriously injured patient would most likely be transferred to the nearest trauma center. In this regard the US may differ from European and even Canadian systems where smaller community or regional hospitals might participate equally in trauma care.

EMP's may also see trauma patients in larger hospitals not designated as trauma centers, with or without formalized trauma systems, but where surgical back-up, surgical specialty care and critical care services are available. There is little if any published data examining this phenomenon though some conclusions might be drawn from the study by MacKensie et al compared outcomes for trauma patients admitted to trauma centers vs. non trauma center hospitals [29,30]. These non-trauma center hospitals averaged more than 200 acute care beds and 19 critical care beds. In this study, 34% of non-trauma center hospitals utilized a trauma team while 66% did not. Thirty-per cent of non-trauma centers utilized in-house surgeons, 70% did not. In this setting it is reasonable to assume that EMP's might evaluate and treat trauma patients but generally require surgical support to admit patients with more serious injuries. Though not specifically supported by the published data, in non-trauma center hospitals lacking formalized trauma admission protocols, EMP's might find that general surgeons and surgical specialists are unwilling to admit patients or accept responsibility for "non-surgical" problems or injuries not in their "body region". Particularly in busy ED's these barriers to admission and disposition become a significant problem for EMP's and a disincentive to participate in trauma care. In some cases EMP's may resort to admitting patients to medical services despite the perceived lack of expertise of those services in managing trauma patients. This practice is perceived as suboptimal care for a trauma patient with serious injuries but again has not been subjected to objective outcome analysis.

Within designated trauma centers in North America the role of EMPs varies considerably depending upon level of trauma center and practice pattern. New roles for EMPs are being defined and studied.

For the most part, in busy trauma centers in the US, EMP's function as members of a trauma team and participate in accordance with ASCOT guidelines. For patients meeting criteria for the highest level of trauma team activation a surgeon must be present and generally acts or is expected to act as the team leader. EMP's often function as the airway manager and/or in other capacities as designated by the team leader (3). These guidelines have implications for training of EM residents who may not have the opportunity to acquire team leadership skills in trauma resuscitation. The requirement that surgeons need to present during trauma activations has been questioned [7,18,31-33].

Within trauma centers there has been consistent interest in designating more than one level of trauma response [34-38]. These efforts have focused upon the implications of the inherent over triage of trauma patients in trauma centers and have utilized EMP's to evaluate and manage those patients. These studies focused upon the impact of over triage on already burdened systems, the cost of full trauma-team activation, and the comparable patient safety achieved using two tiers of trauma response. Interestingly, whether a tiered system is present or not, a proportion of all trauma patients evaluated in any trauma outcome study will be comprised of patients whose evaluation was carried out by EMP's. Discovery of injuries and/or co-morbidities requiring admission will lead to consultation from an admitting trauma service. While this process of care is scrutinized by most well developed trauma program performance improvement programs, the results have not been widely reported in the literature. Virtually all the literature dealing with tiered response reports that triage tools accurately sort patients into low and high acuity groups but there is less detailed evidence regarding process and outcome for severely injured patients inadvertently evaluated in the lower tiered group [34-38]. Examples might include isolated closed head injury or solid organ injury.

Within the past five years several reports have been published in Canada and the USA suggesting that the presence of a surgeon at both adult and pediatric trauma resuscitations is superfluous [7,31-33]. Most of these reports describe the role of the EMP in team leadership during the resuscitative phase of care. Following this ED or resuscitative phase of care, presumably these patients would be admitted to a surgical or trauma service. All the studies referenced report comparable survival based upon the involvement of EMP's during the resuscitation time frame. There has been criticism that these studies suffer from methodological flaws including but not limited to being under powered and containing too few penetrating trauma patients [39]. Nonetheless, these studies serve to quantify what has long been assumed; EMP's can evaluate and treat patients with minor injuries and are able to effectively "team lead" resuscitations for patients with more serious injuries. Whether surgeons should be present in a mandatory or selective fashion remains open to debate but mandatory presence remains standard of care in the US. Selective surgical presence has been validated in Canadian trauma centers where surgical team-leaders are not required [7].

The role of the EMP and the question of expertise in major trauma involving complex decision-making beyond the resuscitative phase of care is less well established by these studies. These components or service elements include but are not limited to coordination of competing clinical priorities such as those related to concomitant neurosurgical and orthopedic emergencies, critical care issues, and diagnosis and treatment of complex problems such as actively bleeding pelvic fractures, high-grade solid organ injury complexes, and major chest trauma.

## Advanced Training Models

Recent publication of data from our group focuses upon the process of advanced training and practice in traumatology by EMP's [40]. The concept of providing fellowship training in Trauma/Critical Care to EMP's is not new. European EMP's may pursue advanced training in Critical Care leading to a specialty board exam. The role of these physicians in trauma care is not well known. In US and Canadian trauma centers this is a relatively new training model but some large centers have used EMP Traumatologists as attending staff and reported favorable results (personal communication). The study by Grossman is the first we know of to report objective data defining process and outcome during the first 24 hours of care [40]. A single fellowship-trained EMP was compared to a group of fellowship-trained Trauma Surgeons. The EMP had trauma surgical back-up available but treated patients independently including driving the decision making process for management of multiple complex injuries after admission to the ICU. The provider groups were similar with respect to several process variables including test ordering, blood transfusion, frequency and severity of patients admitted to ICU. Time to operating room, missed injury and delay in diagnosis were also similar. While this study has many limitations it is the first to evaluate the performance of EMP's who have undergone fellowship training and it examines aspects of care beyond the ED.

EMP's who have received advanced training in Critical Care participate as intensivists managing trauma patients in several large US trauma centers [26,27]. Direct comparison between EMP's and other non-surgical intensivists in the care of trauma patients have not been published to the best of our knowledge. Despite the fact that EMP's who receive advanced training in critical care with or without advanced training in traumatology cannot currently be certified by any US specialty board, some of these physicians have taken the European board exams and utilized their results for credentialing in US hospitals. A novel approach for certification in critical care in the US is additional training in Neuro-intensive care that leads to a certificate of added qualifications offered by the American Board of Medical Specialties.

## Conclusion

The role of EMP's in Trauma care in North America differs considerably from European systems. Trauma care in the US is highly regionalized and has been organized around a surgical model the validity of which has been called into to question by a number of publications in the Emergency Medicine literature (multiple). While there is controversy regarding some of these studies it does seem clear that the current system of care in the US provides effective care for trauma patients. It is equally clear that the system may not be sustainable or desirable in it's current form and in this regard EMP's are likely to have an expanded role in trauma care.

While not proven by objective data it is clear that effective trauma care is a continuum including but not limited to care rendered in the ED. Expertise in trauma care requires understanding of all phases of care whether practiced by Surgeons EMP's or other non-surgeons. Advanced training models for EMP's might allow increased effectiveness of care both inside the ED and beyond it.

## Competing interests

The author declares that they have no competing interests.
